# SMC3 epigenetic silencing regulates Rab27a expression and drives pancreatic cancer progression

**DOI:** 10.1186/s12967-023-04448-1

**Published:** 2023-08-28

**Authors:** Nuno Bastos, Stéphanie A. Castaldo, Bárbara Adem, José C. Machado, Carlos A. Melo, Sonia A. Melo

**Affiliations:** 1 i3S - Instituto de Investigação e Inovação Em Saúde, University of Porto, 4200-135 Porto, Portugal; 2 Institute of Biomedical Sciences Abel Salazar, University of Porto, 4050-313 Porto, Portugal; 3 Department of Pathology, Faculty of Medicine, University of Porto, Rua Alfredo Allen, 208, 4200-135 Porto, Portugal; 4 Porto Comprehensive Cancer Center (P.CCC) Raquel Seruca, Porto, Portugal; 5 The Gurdon Institute and Department of Pathology, University of Cambridge, Cambridge, UK

**Keywords:** Pancreatic cancer, Rab27a, Exosomes, SMC3

## Abstract

**Background:**

Pancreatic ductal adenocarcinoma (PDAC) is expected to soon surpass colorectal cancer as a leading cause of cancer mortality in both males and females in the US, only lagging behind lung cancer. The lethality of PDAC is driven by late diagnosis and inefficient therapies. The complex biology of PDAC involves various cellular components, including exosomes that carry molecular information between cells. Thus, recipient cells can be reprogrammed, impacting tumorigenesis. Rab27a is a GTPase responsible for the last step of exosomes biogenesis. Hence, dissecting the mechanisms that regulate the expression of Rab27a and that control exosomes biogenesis can provide fundamental insights into the molecular underpinnings regulating PDAC progression.

**Methods:**

To assess the mechanism that regulates Rab27a expression in PDAC, we used PDAC cell lines. The biological significance of these findings was validated in PDAC genetically engineered mouse models (GEMMs) and human samples.

**Results:**

In this work we demonstrate in human PDAC samples and GEMMs that Rab27a expression decreases throughout the development of the disease, and that Rab27a knockout promotes disease progression. What is more, we demonstrate that Rab27a expression is epigenetically regulated in PDAC. Treatment with demethylating agents increases Rab27a expression specifically in human PDAC cell lines. We found that SMC3, a component of the cohesin complex, regulates Rab27a expression in PDAC. SMC3 methylation is present in human PDAC specimens and treatment with demethylating agents increases SMC3 expression in human PDAC cell lines. Most importantly, high levels of SMC3 methylation are associated with a worse prognosis in PDAC. Mechanistically, we identified an enhancer region within the Rab27a gene that recruits SMC3, and modulates Rab27a expression.

**Conclusion:**

Overall, we dissected a mechanism that regulates Rab27a expression during PDAC progression and impacts disease prognosis.

**Supplementary Information:**

The online version contains supplementary material available at 10.1186/s12967-023-04448-1.

## Background

Pancreatic ductal adenocarcinoma (PDAC) is an aggressive and lethal malignancy with a 94% mortality rate [1, 2]. The lethality of PDAC is associated with late diagnosis and lack of effective therapeutic options, urging the necessity to better understand PDAC biology [3, 4]. The development and progression of PDAC involves complex cellular interactions and signaling pathways, and recent studies have demonstrated that exosomes play a critical role in cancer progression and metastasis [5–8]. Exosomes are extracellular vesicles of endosomal origin released by cells that contain a variety of bioactive molecules (proteins, lipids, RNA and DNA) and, in cancer, have been shown to mediate intercellular communication and reprogram the tumor microenvironment [5, 6, 9, 10]. One of the main players involved in exosomes biogenesis and secretion is the Rab GTPase family of proteins. Rab GTPases regulate vesicle trafficking and membrane fusion events in cells, including exosomes release [11]. Among the many Rab GTPases, Rab27a has been shown to regulate the late stages of the endocytic pathway mediating exosomes release from cancer cells, including in PDAC [10, 12]. Several studies have demonstrated that Rab27a is critical for efficient exosomes-mediated communication modulating different cancer related processes such as cancer cells proliferation and dissemination, as well as remodeling of the tumor microenvironment [13–15]. Understanding the regulation of expression of Rab GTPases, such as Rab27a, and their impact on exosomes biogenesis and secretion throughout cancer progression could provide important insights into the biology of PDAC and unravel novel opportunities for the development of targeted therapies aimed at modulating intercellular communication mediated by exosomes. Here we dissected the mechanism that regulates Rab27a expression in PDAC and that may condition communication mediated by exosomes during the course of the disease.

## Results

### Rab27a protein expression decreases throughout PDAC progression

**Fig. 1:**
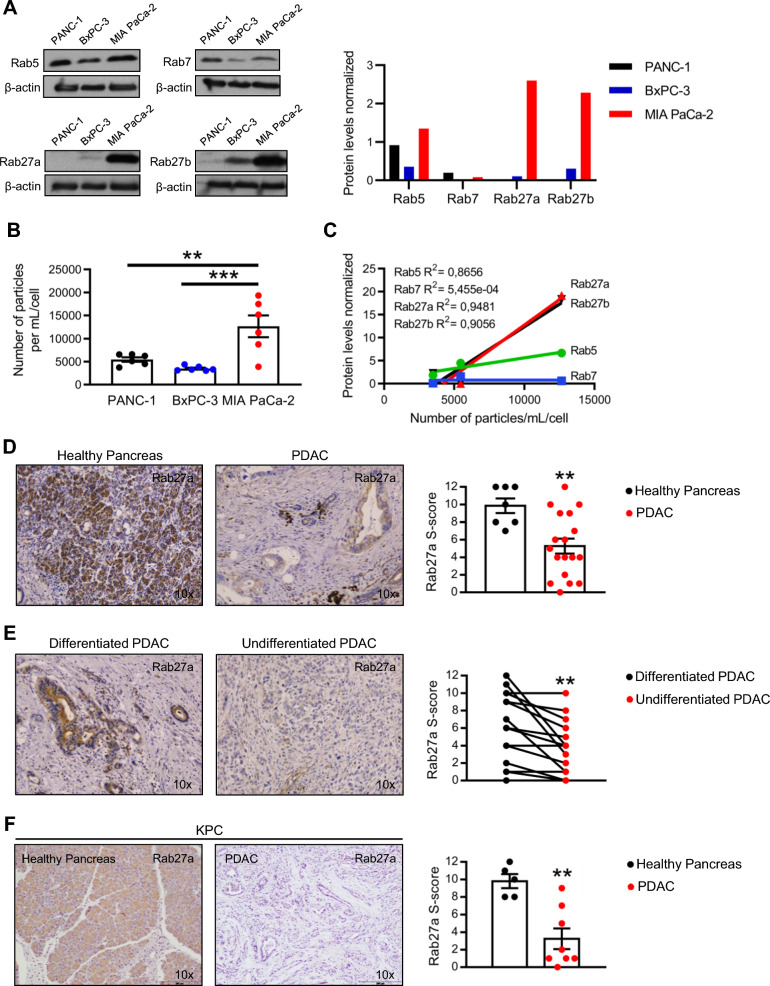
Rab27a protein levels correlate the best with the number of extracellular vesicles released in PDAC and its expression decreases throughout disease progression. **A** Western blot of Rab5, Rab7, Rab27a and Rab27b (left) and respective quantification (right) in PANC-1, BxPC-3 and MIA PaCa-2 cells. β-actin was used as loading control. **B** Number of particles per cell and per mL released by PANC-1, BxPC-3 and MIA PaCa-2 cells (n = 6). One-way anova **p < 0.01, ***p < 0.001. **C** Correlation between the normalized Rab5 (green), Rab7 (blue), Rab27a (red) and Rab27b (black) protein levels and the number of particles secreted per cell and per mL in PANC-1, BxPC-3 and MIA PaCa-2 cells. Linear regression. **D** Representative Rab27a IHC images (10x, left) and quantification (right) in histologically healthy pancreas (n = 7) and PDAC (n = 18) tissues from human specimens (Unpaired t-test ** p < 0.01). **E** Representative Rab27a IHC images (10x, left) and quantification (right) in differentiated and undifferentiated PDAC tissues from the same human specimen (n = 15, Paired t-test ** p < 0.01). **F** Representative Rab27a IHC images in histologically healthy pancreas (n = 5) and PDAC (n = 8) in KPC mice (left, 10x) and quantification (right). Unpaired t-test **p < 0.01. Data are Mean ± SEM

In order to determine which is the best Rab GTPase to analyze as a surrogate marker for secretion of exosomes by cancer cells, we screened for four Rab GTPases (Rab5, Rab7, Rab27a and Rab27b) using three human PDAC cell lines (PANC-1, BxPC-3 and MIA PaCa-2). Each Rab GTPase corresponds to a specific step in exosomes biogenesis, from early endosomes to exocytosis of exosomes. We demonstrated that all Rabs are differentially expressed between the PDAC cell lines analyzed, being this difference more striking for Rab27a and Rab27b (Fig. 1A). MIA PaCa-2 is the human PDAC cell line that expresses the higher amounts of Rab27a and 27b proteins (Fig. 1A). As expected, and in agreement with the Rab27a and 27b expression levels, MIA PaCa-2 is also the cell line that secretes the highest number of vesicles in comparison with PANC-1 and BxPC-3 (Fig. 1B). Most importantly, amongst the four Rab GTPases analyzed, Rab27a protein levels is the one that correlates the best with the number of secreted vesicles by cancer cells in a linear regression analysis (Fig. 1C). In addition, previous work also demonstrates that Rab27a expression is important for exosomes secretion [12, 13, 15]. Based on our results and previously published data, we have focused our analysis on Rab27a and Rab27a-dependent exosomes communication in PDAC.

We have started by evaluating Rab27a protein expression by immunohistochemistry (IHC) during PDAC progression in a cohort of human PDAC samples (n = 18). We observed a significant decrease in Rab27a protein levels in PDAC tissue in comparison to adjacent healthy pancreas (Fig. 1D). The same holds true when comparing differentiated to undifferentiated tumor lesions (Fig. 1E). Most importantly, we have recapitulated the results observed in human samples using the KPC mouse model (*LSL-Kras*^*G12D/*+^; *LSL-Tp53*^*R172H/*+^; *Pdx1-Cre*), a genetically engineered mouse model (GEMM) that spontaneously develops PDAC (Fig. 1F) [16]. We demonstrate that PDAC tissue in KPC mice show significantly lower expression levels of Rab27a in comparison to healthy pancreas (Fig. 1F).

Taken together, using human PDAC samples and a well-established PDAC GEMM we demonstrated that Rab27a protein expression is lost during the course of the disease.

### Rab27a behaves as a tumor suppressor gene in PDAC and its expression is epigenetically regulated

**Fig. 2:**
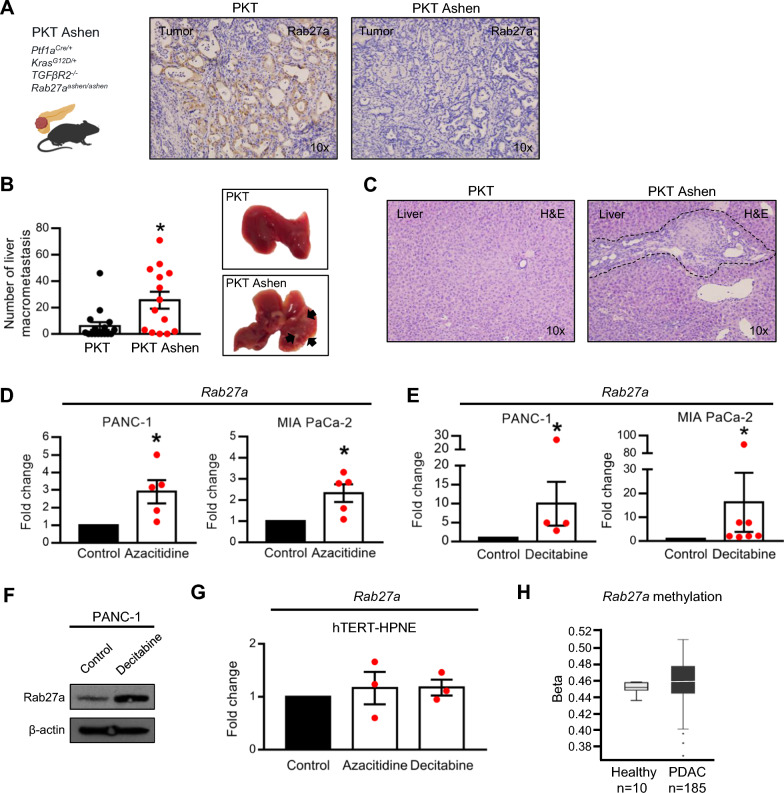
Rab27a expression is regulated via an indirect epigenetic mechanism. **A** Representative Rab27a IHC in PKT and PKT Ashen tumors (10x). **B** Representative liver images (right) and number of liver macrometastasis (left) in PKT (n = 16) and PKT Ashen (n = 14) mice. Arrows indicate sites of metastasis. Unpaired t-test *p < 0.05. **C** Representative liver H&E of PKT and PKT Ashen mice (10x). Dashed lines indicate metastatic lesion. **D** Fold change of Rab27a expression determined by qPCR in PANC-1 and MIA PaCa-2 treated with azacitidine (5 μM—PANC-1, 1 μM—MIA PaCa-2). N = 5, unpaired t-test, *p < 0.05. **E** Fold change of Rab27a expression determined by qPCR in PANC-1 and MIA PaCa-2 treated with decitabine (5 μM—PANC-1, 1 μM—MIA PaCa-2). N = 4 in PANC-1, n = 7 in MIA PaCa-2, unpaired t-test, *p < 0.05. **F** Western blot of Rab27a in PANC-1 treated with vehicle (control) or decitabine (5 μM). β-actin was used as loading control. **G** Fold change of Rab27a expression determined by qPCR in hPNE-hTERT treated with azacitidine (1 μM) or decitabine (1 μM) (n = 3). **H** Rab27a DNA methylation beta values in healthy pancreas and PDAC in human samples. Data extracted from DNMIVD platform. Data are Mean ± SEM

In order to better understand the role of Rab27a in PDAC development and progression, we generated the PKT Ashen model. The PKT (*Ptf1a-Cre; LSL-Kras*^*G12D/*+^; *Tgfbr2*^*loxP/loxP*^*)* is a GEMM that develops PDAC in a spontaneous manner, recapitulating the different stages of the human disease [17]. This is a fast progression model with a more homogeneous progression of the disease when compared to the KPC, which takes several months to develop PDAC. In combination with the Ashen mouse, a constitutional Rab27a knockout, we obtain a GEMM that develops PDAC in a Rab27a knockout background (PKT Ashen, Fig. 2A) [18]. Rab27a knockout was validated by immunohistochemistry (IHC) in tumors of PKT Ashen mice (Fig. 2A). Interestingly, we observed a significant increase in tumor burden in the PKT Ashen (Rab27a KO) in comparison with the PKT (Rab27a WT) reflected in a significant increase in the number of liver macrometastasis in the Rab27 KO mice (Fig. 2B, C). These results indicate that loss of Rab27a expression in cancer cells represents an advantage for disease progression in PDAC.

Expression of Rab27a in human and mouse samples decreases during the course of the disease. In addition, loss of Rab27a expression accelerates disease in a PDAC GEMM. In sum, Rab27a gene behaves as a tumor suppressor in PDAC. Therefore, we went on to look for the mechanism that regulates Rab27a expression in these tumors. Genetic and epigenetic mechanisms are the top candidate processes to regulate gene expression. We have queried the TCGA (Cancer Genome Atlas Program) for somatic mutations across the Rab27a gene in human PDAC samples and we have not identified any mutations that could explain the observed loss of expression of Rab27a in PDAC. Therefore, we went on and tested if epigenetic regulation could be the mechanism by which Rab27a expression is lost in PDAC. Methylation is frequently observed in human cancer and targets many genes involved in the progression of the disease. To test this hypothesis, we treated two human PDAC cell lines (PANC-1 and MIA PaCa-2) with the demethylating agents, azacitidine and decitabine. Rab27a expression levels significantly increase in cancer cells treated with any of the two demethylating agents (Fig. 2D, E). In addition, an increase in Rab27a protein levels was validated in PANC-1 cells treated with decitabine (Fig. 2F). In stark contrast, treatments with the same demethylating agents did not alter Rab27a expression in a healthy pancreas cell line (hTERT-HPNE, Fig. 2G). Hence, our data shows that an epigenetic regulatory mechanism of Rab27a expression occurs specifically in cancer. We went on and interrogated the DNMIVD platform for Rab27a methylation levels in human PDAC tissue (n = 185) and healthy pancreas (n = 10) [19]. The average levels of methylation across the Rab27a gene were not significantly different between PDAC patients and healthy tissue (Fig. 2H). Therefore, we could conclude that Rab27a expression is indirectly regulated by an epigenetic-driven mechanism.

### Epigenetic regulation of SMC3 modulates Rab27a expression in PDAC

**Fig. 3:**
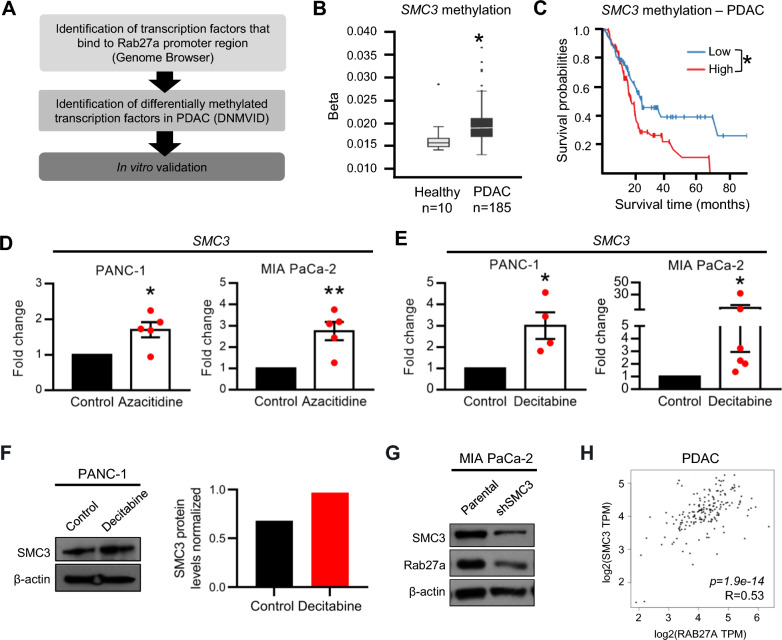
SMC3 methylation is associated with worse prognosis in PDAC and its expression correlates with Rab27a expression. **A** Pipeline to determine potential targets that regulate Rab27a expression in PDAC. **B** SMC3 DNA methylation beta values in healthy pancreas and PDAC in human samples. Data extracted from DNMIVD platform. Unpaired t-test, *p < 0,05. **C** Kaplan–Meier curve of PDAC patients with low (blue) or high (red) levels of SMC3 methylation. Data extracted from DNMIVD platform. Log-rank test, *p < 0.05. **D** Fold change of SMC3 expression determined by qPCR in PANC-1 and MIA PaCa-2 treated with azacitidine (5 μM – PANC-1, 1 μM – MIA PaCa-2). N = 5, unpaired t-test, *p < 0.05, **p < 0.01. **E** Fold change of SMC3 expression determined by qPCR in PANC-1 and MIA PaCa-2 treated with decitabine (5 μM—PANC-1, 1 μM—MIA PaCa-2). N = 4 in PANC-1, n = 6 in MIA PaCa-2, unpaired t-test, *p < 0.05. **F** Western blot of SMC3 (left) and respective quantification (right) in PANC-1 treated with vehicle (control) or decitabine (5 μM). β-actin was used as loading control. **G** Western blot of SMC3 and Rab27a in MIA PaCa-2 parental cell line or transfected with a short hairpin that targets SMC3. β-actin was used as loading control. **H** RNA expression correlation of SMC3 and Rab27a in human PDAC samples. R = 0.53, p = 1.9 × 10^–14^. Data extracted from TCGA. Data are Mean ± SEM

We next tested the hypothesis that epigenetic regulation of transcription factors that bind Rab27a gene could control its expression and explain our observations. Thus, we screened the genome browser to identify transcription factors that bind the promoter region of Rab27a. We have also filtered for those transcription factors that, besides binding to Rab27a, are also methylated in human PDAC samples (Fig. 3A). SMC3 emerged as one of the best candidates because it has two binding regions in the Rab27a gene (one in the promoter region and a second one next to a potential enhancer region). SMC3 is part of the cohesin complex, which mediates sister chromatid cohesion, double-stranded DNA break repair and regulates gene expression [20]. We demonstrate that SMC3 methylation levels are increased in PDAC tissue in comparison to healthy pancreas using the DNMIVD platform (Fig. 3B) [19]. Most importantly, high levels of SMC3 methylation are associated with worse prognosis in PDAC (Fig. 3C) [19]. Thus, we went on to validate that SMC3 is regulated by methylation and determine if the expression of Rab27a is dependent on the expression of SMC3. In agreement with our previous observations, treatment with demethylating agents, azacitidine and decitabine, led to a significant increase in SMC3 expression and protein levels in PDAC cell lines, suggesting that this gene is commonly methylated in PDAC (Fig. 3D–F). Next, to understand if Rab27a expression is dependent on SMC3, we transfected MIA PaCa-2 cells with a short hairpin RNA (shRNA) targeting SMC3 and evaluated Rab27a protein levels. Upon SMC3 knockdown we observed a decrease in Rab27a protein levels suggesting that Rab27a could be regulated by SMC3 (Fig. 3G). Notably, SMC3 expression levels positively correlate with Rab27a expression in human PDAC samples (analysis of TCGA data using the GEPIA platform; Fig. 3H) [21].

### SMC3 mediates Rab27a expression by supporting promoter-enhancer interactions

**Fig. 4:**
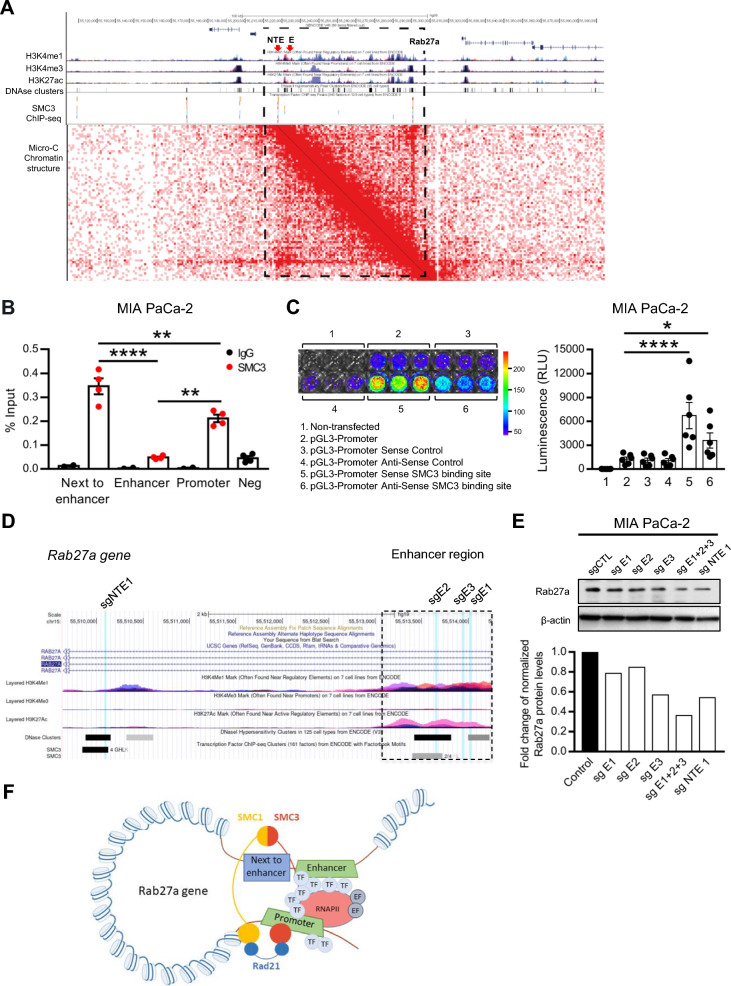
SMC3 binding to Rab27a gene is crucial for gene expression. **A** Hi-C dataset analysis for the study of the binding of SMC3 protein in the Rab27a gene using the H1-hESC cell line. Dashed lines highlight the region of the Rab27a gene. **B** ChIP-qPCR of SMC3 for different binding sites in the Rab27a gene (Promoter, Enhancer and Next to Enhancer regions) in MIA PaCa-2 cells (n = 4). IgG antibody was used as control so that we could identify regions of specific binding for Rab27a. Negative region for SMC3 binding was used to confirm specificity of the enrichment (Neg). One-way anova **p < 0.01, ****p < 0.0001. **C** Luminescent imaging (left) and quantification (right) of MIA PaCa-2 cells non-transfected (1), transfected with pGL3-Promoter empty vector (2), pGL3-Promoter vector with a control sequence (sense, 3), pGL3-Promoter vector with a control sequence (anti-sense, 4), pGL3-Promoter vector with SMC3 binding site in the enhancer region of Rab27a gene (sense, 5) or pGL3-Promoter vector with SMC3 binding site in the enhancer region of Rab27a gene (anti-sense, 6). N = 6, two-way anova, *p < 0.05, ****p < 0.0001. **D** Genome browser panel highlighting regions in the Rab27a gene targeted by sgE1, sgE2, sgE3 and sg NTE1. Sg1 and 3 target regions of the Rab27a enhancer. SgE2 targets SMC3 binding site in the Rab27a enhancer region and sgNTE1 targets SMC3 binding site in a region next to the Rab27a enhancer. Dashed lines identify the enhancer region in the Rab27a gene. **E** Western blot of Rab27a (top) and quantification (bottom) in MIA PaCa-2 cells transduced with a control sg (intron region of Rab27a gene), a sg for Rab27a enhancer region 1, a sg for Rab27a enhancer region 2, a sg for Rab27a enhancer region 3, a combination of sgs for different regions of the Rab27a enhancer (1 + 2 + 3) or a sg for next to enhancer region 1. β-actin was used as loading control. **F** Schematic representation of the proposed mechanism of SMC3 binding to the Rab27a gene. Data are Mean ± SEM

SMC3 within the cohesin complex regulates gene expression through 3D genome organization facilitating promoter and enhancer contacts [22]. Using the UCSC genome browser chromatin-immunoprecipitation sequencing (ChIP-seq) tracks for SMC3, we verified that SMC3 is able to bind the promoter region of Rab27a gene as well as two other regions within close proximity (Fig. 4A) [23]. Based on the presence of high histone 3 lysine 4 mono-methylation and histone 3 lysine 27 acetylation marks present in one of the regions, we defined these locations as enhancer (E) and next to enhancer (NTE) regions (Fig. 4A). We postulated that NTE serves as an anchor region to bring Rab27a enhancer and promoter regions together. Indeed, we have used available databases to study in situ chromatin interactions by means of Hi-C chromatin structure analysis [24]. We showed that NTE, where SMC3 binds with high affinity, contacts with the promoter region of Rab27a (Fig. 4A). To support our hypothesis, we validated through ChIP-qPCR the binding of SMC3 to these three regions. In agreement with what is observed in SMC3 ChIP-seq datasets, we observed high SMC3 binding to the promoter and NTE regions in a human PDAC cell line (Fig. 4B). In order to confirm that the region defined as an enhancer, and specifically the SMC3 binding site, has the ability to promote gene expression, we cloned this sequence into a pGL3-Promoter luciferase reporter plasmid. Strikingly, both the sense and anti-sense sequences of the SMC3 binding site in the enhancer region led to an increase in the luminescence signal, indicating that these sequences have enhancer function (Fig. 4C). Next, to determine the impact on Rab27a expression of the enhancer and NTE regions, we designed different CRISPR sgRNA sequences to target both regions (Fig. 4D). Small-guided RNA sequences E1, E2 and E3 were designed to target the enhancer region of Rab27a (Fig. 4D). Additionally, sgRNA sequence NTE1 was designed to target the SMC3 binding site in the NTE region (Fig. 4D). We show that all sgRNA sequences led to a decrease in Rab27a protein levels (Fig. 4E). The combination of three sgRNA sequences targeting the enhancer region and the single sgRNA targeting the NTE region were the conditions that most impacted Rab27a protein levels (Fig. 4E). These results demonstrate that the enhancer and the NTE regions are important for Rab27a expression. In addition, the NTE region also appears to be a crucial anchor point, through a SMC3-dependent spatial gene organization mechanism (Fig. 4F).

## Discussion

Our work focused on understanding how Rab27a expression changes throughout the different stages of PDAC progression and how this expression is regulated. We described that Rab27a expression decreases upon malignant transformation and is further silenced in undifferentiated PDAC lesions. More importantly, impairment of Rab27a expression leads to an increase in metastatic liver burden in a PDAC GEMM. We identified a novel epigenetic mechanism mediated by SMC3 that regulates Rab27a expression. Methylation of SMC3 inhibits its binding to Rab27a resulting in gene silencing, driving disease progression.

Progressive loss of gene expression from healthy pancreas to undifferentiated cancer tissue suggests a tumor suppressive role for Rab27a. In addition, we demonstrate that in a Rab27a knockout context an increase in the metastatic burden occurs in PDAC GEMMs, which further strengthens this observation. Thus far, the majority of reports associate Rab27a and exosomes mediated communication with an oncogenic function by promoting tumor growth and dissemination [8, 14, 15]. However, the majority of mouse studies were performed using orthotopic models in immunodeficient backgrounds. Interestingly, a report on pancreatic cancer demonstrated for the first time a dual role for Rab27a in the metastatic potential of cancer cells [25]. Loss of Rab27a compromises efficient outgrowth of pancreatic cancer metastatic lesions, however, it also provides an advantage at the first steps of metastasis establishment, through upregulation of different genes associated with epithelial-to-mesenchymal transition [25]. Together with our findings, this indicates that in a setting of natural development of the disease loss of Rab27a expression could be more advantageous rather than detrimental in the metastatic process. However, since the Ashen mouse is a constitutional Rab27a knockout, we cannot exclude a role of Rab27a impairment in other cells of the organism in PDAC progression.

Rab27a mutations have not been described, thus far, in the context of cancer. In fact, when performing TCGA data analysis in PDAC samples we only identified one somatic mutation in one sample which further indicates that epigenetic regulation could play a role in Rab27a expression. Here, we focused on a mechanism mediated by methylation since upon treatment with demethylating agents we observed an increase in Rab27a expression in PDAC cell lines. Nonetheless, Rab27a expression could also be regulated by additional mechanisms. Several studies have demonstrated the ability of non-coding RNAs, in particular microRNAs (miRNAs) and long non-coding RNAs, in controlling gene expression [26]. In fact, different miRNAs such as, miR-134-3p, miR-124 and miR-582-5p have been demonstrated to impair Rab27a expression in different models [27–29]. The study of these non-coding RNAs and others could also be relevant to better understand how modulation of exosomes communication occurs in PDAC.

We propose and have shown it using Hi-C data, that SMC3 binds to the Rab27a gene preferentially in the promoter and next to enhancer regions conferring a conformational change that facilitates interaction between the promoter and enhancer driving transcriptional activity.

## Conclusion

In sum, we describe an epigenetic mechanism that regulates Rab27a expression indirectly through SMC3. We have for the first time described an enhancer region within the Rab27a gene. Based on our findings, this mechanism can promote disease progression in the context of PDAC. Targeting of SMC3 expression and activity could potentially be used to modulate Rab27a-dependent exosomes communication and improve patients’ outcome.

## Methods

### Patient tissue samples

Tumor paraffin blocks from PDAC patients were obtained from *Centro Hospitalar Tondela-Viseu*, Viseu, Portugal and from *Carl Gustav Carus University Hospital, Dresden, Germany*. Informed consent was required from all patients. Clinical information provided included age, sex and tumor stage.

### Mice

KPC (*Pdx1-Cre; LSL-Kras*^G12D/+^*; LSL-Trp53*^R172H/+^) and PKT (*Ptf1a-Cre*; *LSL-Kras*^G12D/+^*; Tgfbr2*^*loxP/loxP*^) mouse models alleles were purchased from Jackson Laboratory. B6.FVB-Tg(Pdx1-Cre)6Tuv/J *(*RRID:IMSR_JAX:014647*)*; B6.129S4-Krastm4Tyj/J (RRID:IMSR_JAX:008179); 129S-Trp53tm2Tyj/J (RRID:IMSR_JAX:008652); Ptf1atm1(Cre)Hnak/RschJ (RRID:IMSR_JAX:023329); B6;129-Tgfbr2tm1Karl/J (RRID:IMSR_JAX:012603).

*Rab27a*^*ash/ash*^ allele was kindly provided by Doctor Miguel Seabra, CEDOC, NOVA Medical School, Lisbon, Portugal [18].

PKT Ashen (*Ptf1a-Cre*; *LSL-Kras*^G12D/+^*; Tgfbr2*^*loxP/loxP*^; *Rab27a*^*ash/ash*^) developed PDAC in a spontaneous manner in a similar way to the PKT mouse model.

Regarding the KPC mouse model, a cross-sectional study was performed. Mice were euthanized at different timepoints of disease progression (8 weeks, 16 weeks and HEP - humane end point). PKT and PKT Ashen mice were euthanized when presented severe symptoms.

All mice were housed under standard housing conditions at the i3S animal facility.

### Immunohistochemistry

4 μm sections were used for immunohistochemistry staining. Prior to antibody incubation, heat-mediated antigen retrieval was performed for 40 min using a citrate buffer pH 6 solution (Vector Laboratories) followed by an incubation for 30 min at RT with a protein block solution (Dako). Overnight incubation at 4ºC was performed for the primary antibody anti-Rab27a 1:200 (Sigma-Aldrich Cat# HPA001333, RRID:AB_1079730). After washing steps, incubation for 30 min at RT with anti- Rabbit/Mouse HRP Dako REAL EnVision Detection System, Peroxidase/DAB (Dako) was performed. Finally, incubation with DAB solution was performed for 1 min. Rab27a score in mouse and human samples was performed based on staining intensity and the percentage of cells present for each intensity. Score 0, 1, 2 or 3 corresponds to negative, weak, intermediate or strong staining, respectively. The percentage of cells for each staining intensity within the slide was also associated with a score, 1—0–25%, 2–25–50%, 3–50–75%, 4- > 75%. The final formula applied was: Rab27a score = Score intensity x Area percentage score. A score between 0–12 was given to each tumor slide.

### Cell culture

The following cell lines were used: MIA PaCa-2 (ATCC Cat# CRL-1420, RRID:CVCL_0428), PANC-1 (ATCC Cat# CRL-1469, RRID:CVCL_0480), BxPC-3 (ATCC Cat# CRL-1687, RRID:CVCL_0186), hTERT-HPNE (ATCC Cat# CRL-4023, RRID:CVCL_C466) and HEK293T (ATCC Cat# CRL-3216, RRID:CVCL_0063).

All cells were tested for mycoplasma and STR profiled for our study. All PDAC cell lines (MIA PaCa-2, PANC-1 and BxPC-3) and HEK293T were cultured in RPMI-1640 medium (Gibco) supplemented with 10% (v/v) fetal bovine serum (FBS, Gibco), 100 U/mL penicillin and 100 μg/mL streptomycin (Gibco). hTERT-HPNE was cultured in RPMI-1640 medium supplemented with 20% (v/v) FBS, 100 U/mL penicillin and 100 μg/mL streptomycin. Cell lines were cultured at 5% CO_2_ and 37 °C in a humidified incubator.

### Azacitidine and decitabine treatments

2 × 10^5^ cells of MIA PaCa-2, PANC-1 and hTERT-HPNE were plated in 6-well plates. On the next day, cells were washed and treated with either azacitidine (Sigma-Aldrich) or decitabine (Sigma-Aldrich) for the following 96 h. During the treatment period, cells were washed daily and fresh medium containing the appropriate concentration of the demethylating agent was used. In azacitidine (Sigma-Aldrich) and decitabine (Sigma-Aldrich) treatments, PANC-1 were treated with a final concentration of 5 μM and MIA PaCa-2 and hTERT-HPNE were treated with a final concentration of 1 μM. Vehicle-treated cells (water/acetic acid 1:1 v/v) were used as control for both azacitidine (Sigma-Aldrich) and decitabine (Sigma-Aldrich) treatments. Technical triplicates were used in each experiment.

### Quantitative reverse transcription-PCR

RNA isolation was performed using TRIzol (Thermo Fisher Scientific) as described in the manual. cDNA synthesis was performed using the NZY First-Strand cDNA Synthesis Kit (NZYTech) according to the manufacturer´s instructions. For qPCR analysis, Power SYBR green PCR master mix (Applied Biosystems) was used.

Oligonucleotide sequences used for quantification were:

Rab27a forward: AGCTTTGGGAGACTCTGGTG.

Rab27a reverse: TGTGTCCCATAACTGCAGGT.

SMC3 forward: GGAGGGCAGTCAGTCTCAAG.

SMC3 reverse: AGCAAGGGCTACCAAGGATT.

β-actin forward: GAGCACAGAGCCTCGCCTTT.

β-actin reverse: ACATGCCGGAGCCGTTGTC.

2^−∆∆CT^ method was used to calculate the fold change between conditions.

### ChIP-qPCR

Cross-linking ChIP in MIA PaCa-2 was performed using 5 × 10^6^ cells per immunoprecipitation. Cells were fixed using 1% formaldehyde for 10 min at room temperature with gentle shaking and, afterwards, glycine was added for quenching (125 mM, incubated for 2 min at room temperature). Then, cells were washed twice with cold PBS 1X, prior to scrapping.

To obtain a soluble chromatin extract, cells were resuspended in 10 mL LB1 (50 mM HEPES–KOH, pH 7.5; 140 mM NaCl; 1 mM EDTA; 10% glycerol; 0.5% NP-40; 0.25% Triton X-100; 1 × Complete protease inhibitor) and incubated in rotation at 4 °C for 10 min. Samples were then centrifuged and resuspended in 10 mL LB2 (10 mM Tris–HCl, pH 8; 200 mM NaCl; 1 mM EDTA; 0.5 mM EGTA; 1 × Complete protease inhibitor), and incubated in rotation at 4 °C for 5 min. Finally, samples were centrifuged and resuspended in 3 mL LB3 (10 mM Tris–HCl, pH 8; 100 mM NaCl; 1 mM EDTA; 0.5 mM EGTA; 0.1% Na-deoxycholate, 0.5% N-lauroylsarcosine; 1 × Complete protease inhibitor). Chromatin extracts were sonicated for 18 cycles of 30 s ON/OFF, at high power with a Diagenode Bioruptor Plus sonicator. 1% Triton X-100 was added to the sonicated lysates and 1% was kept as whole-cell extract (WCE).

The lysates were incubated with 8 μg anti-SMC3 (Abcam Cat# ab9263, RRID:AB_307122) or 2 μg anti-rabbit IgG (Thermo Fisher Scientific Cat# 10500C, RRID:AB_2532981) antibodies bound to 100 μl protein G Dynabeads (Invitrogen) and incubated overnight at 4 °C. Magnetic beads were washed with RIPA buffer (140 mM NaCl; 0.1% SDS; 1% Triton X-100; 1 mM EDTA; 0.5 mM EGTA; 10 mM Tris–HCl, pH 8; 0.1% sodium deoxycholate) for 4 to 5 times, then washed with TBS. After centrifugation, DNA was eluted (Tris–EDTA, pH 8) and reverse cross-linked (200 mM NaCl; 0.5% SDS and Proteinase K) for at least 5 h at 65 °C. The eluate was then treated with RNase for 30 min at 37 °C. Finally, DNA was cleaned and purified with Zymo ChIP Clean and concentrator kit (Zymo Research).

ChIP-qPCR was performed using Power SYBR green PCR master mix (Applied Biosystems). Primers sequences used were as follows:

SMC3 promoter region.

Forward—CCTCTGTCGGAAGAAACCTG.

Reverse—GAACTTGGCTGCCTCTGAGT.

SMC3 enhancer region.

Forward—TGGTTTCCATTGCTTCATCA.

Reverse—TACCGGCCAGTCTGAAATGT.

SMC3 Next to enhancer region.

Forward—CAAGAGGATGTATTGTTCCCATT.

Reverse—TCCAAATGGCCTTTAAGTGG.

SMC3 Negative region.

Forward—TGCCATGCGTTGAAAATATCC.

Reverse—TGCTTTCTGAAGTTGCCAAGC.

% Input was calculated by dividing the enrichment of each region in SMC3-IP samples by the WCE. Negative control region was used to validate the specificity of the enrichment in the analyzed regions.

### CRISPR

1 × 10^6^ HEK293T cells were plated in a T25 flask for viral particles production. On the next day, cells were washed and transfected with the plasmids psPAX2 packaging (RRID:Addgene_12260), VSV/G envelope (RRID:Addgene_8454) and lentiCRISPR v2 (RRID:Addgene_52961) containing the sgRNA sequence of interest using Lipofectamine 2000 (Thermo Fisher Scientific) using the manufacturer´s instructions. After 72 h, cell culture medium containing viral particles was centrifuged at 2000 g for 10 min to remove cell debris and consequently filtered through a 200 nm strainer (GE Healthcare). Then, the resultant culture medium supplemented with polybrene (10 µg/mL) was used to culture MIA PaCa-2 cells previously plated in 6-well plates. After infection, puromycin (1 μg/mL, Sigma-Aldrich) was used to enrich the cell culture in transduced cells.

Sg sequences used were as follows:

Control: GGATCAGAGTCAAGAATACGTGG.

Enhancer 1: TTGTCACAGGGCTAACAACATC.

Enhancer 2: GAGGCACTCATGTAACGTAGTGG.

Enhancer 3: CAGATAAGCGACAATATATGAGG.

Next to enhancer 1: TGGAAATGCATTATAATTAA.

### Transfection and luminescence assay

2.5 × 10^5^ MIA PaCa-2 cells were plated in 6-well plates. On the following day, cells were either non-transfected or transfected with shSMC3 in a pLKO1 vector (TRCN0000160156, sh sequence—CGAGTAGAGACTTATCTCAAT). After 72 h, cells were harvested and lysed for western blot.

5 × 10^4^ MIA PaCa-2 cells were plated in 24-well plates. On the next day, cells were either non-transfected or transfected with empty pGL3-Promoter plasmid (Promega, Cat # E1761), pGL3-Promoter Sense Control, pGL3-Promoter Anti-Sense Control, pGL3-Promoter Sense SMC3 binding site, pGL3-Promoter Anti-Sense SMC3 binding site using Lipofectamine 2000 (Thermo Fisher Scientific) using the manufacturer´s instructions. Sequences used to clone upstream of the promoter of pGL3-Promoter plasmid are described in Additional file 1: Table S1. Cloning was done in collaboration with GenScript.

After 48 h, cells were harvested and plated in triplicates in 96-well plates. On the following day, Dual-Luciferase^®^ Reporter Assay System (Promega) was used according to the manual to detect luciferase activity. To detect luminescence signal Synergy Mx (BioTek) was used.

Representative picture of luciferase activity using IVIS Lumina iii (Perkin Elmer IVIS Spectrum In-Vivo Imaging System (RRID:SCR_020397)) was taken. Luminescent image mode was used to detect luminescence signal. Luminescence intensity is depicted by a multicolor scale ranging from blue (least intense) to red (most intense).

### Western blot

Western blot was performed as described previously [13]. 30 µg of protein derived from cell lines was used. Primary antibody incubation was performed overnight at 4 ºC. Antibody dilutions used were: anti-Rab27a 1:500 (Abnova Cat# H00005873-M02, RRID:AB_519010), anti-SMC3 1:1000 (Abcam Cat# ab9263, RRID:AB_307122). After washing steps, membranes were incubated with the respective HRP-conjugated secondary antibody at 1:5000 dilution for 1 h at RT (Anti-mouse IgG HRP-linked antibody Advansta Cat# R-05071–500, RRID:AB_10718209; Anti-rabbit IgG HRP-linked antibody Cell Signaling Technology Cat# 7074, RRID:AB_2099233). β-actin (Sigma-Aldrich Cat# A3854, RRID:AB_262011) was used for loading control.

### Exosomes isolation

Exosomes isolation was performed as previously described [13]. Succinctly, PDAC cells were cultured in RPMI medium with exosomes-free FBS for 72 h. Afterwards, medium was centrifuged at 2500RPM for 10 min followed by a centrifugation at 4000RPM for 5 min to remove cell debris. Next, medium was filtered using a 200 nm filter (GE Healthcare) and ultracentrifuged at 100000 g for 3 h at 4 ºC. Finally, supernatant was discarded and exosomes pellet was resuspended in 100 µL of PBS 1X for NanoSight NS300 (Nanoparticle Tracking Analysis, RRID:SCR_014239) analysis.

### Statistical analysis

For the analyses performed in this manuscript significance was determined at *p* < 0.05*,* p* < 0.01**,* p* < 0.001***, *p* < 0.0001**** and represent significance between conditions. All analyses were performed using GraphPad Prism^®^ (GraphPad Prism, RRID:SCR_002798).

### Supplementary Information

**Additional file 1:** **Table S1.** pGL3-promoter sequences for luminescence assay.

## Data Availability

The source data file contains all the source data related to the manuscript and is deposited in the Figshare Data Repository DOI: 10.6084/m9.figshare.23799390.
